# The production and sales of anti-tuberculosis drugs in China

**DOI:** 10.1186/s40249-016-0184-z

**Published:** 2016-10-04

**Authors:** Yang-Mu Huang, Qi-Peng Zhao, Qiao-Meng Ren, Dan-Lu Peng, Yan Guo

**Affiliations:** School of Public Health, Peking University, 38 Xueyuan Road, Haidian District, Beijing, 100191 China

**Keywords:** Tuberculosis, Drug, First-line drug, Second-line drug, Rifampicin, China, Production, Sales

## Abstract

**Background:**

Tuberculosis (TB) is a major infectious disease globally. Adequate and proper use of anti-TB drugs is essential for TB control. This study aims to study China’s production capacity and sales situation of anti-TB drugs, and to further discuss the potential for China to contribute to global TB control.

**Methods:**

The production data of anti-TB drugs in China from 2011 to 2013 and the sales data from 2010 to 2014 were extracted from Ministry of Industry and Information Technology database of China and IMS Health database, respectively. The number of drugs was standardized to the molecular level of the key components before calculating. All data were described and analyzed by Microsoft Excel.

**Results:**

First-line drugs were the majority in both sales (89.5 %) and production (92.3 %) of anti-TB drugs in China. The production of rifampicin held the majority share in active pharmaceutical ingredients (APIs) and finished products, whilst ethambutol and pyrazinamide were the top two sales in finished products. Fixed-dose combinations only held small percentages in total production and sales weight, though a slight increase was observed. The production and sales of streptomycin showed a tendency of decrease after 2012. The trends and proportion of different anti-TB drugs were similar in production and sales, however, the production weight was much larger than that of sales, especially for rifampicin and isoniazid.

**Conclusions:**

First-line drugs were the predominant medicine produced and used in China. While the low production and sales of the second-line TB drugs and FDCs rose concerns for the treatment of multiple drug resistant TB. The redundant production amount, as well as the prompt influence of national policy on drug production and sales, indicated the potential for China to better contribute to global TB control.

**Electronic supplementary material:**

The online version of this article (doi:10.1186/s40249-016-0184-z) contains supplementary material, which is available to authorized users.

## Multilingual abstracts

Please see Additional file 1 for translations of the abstract into the six official working languages of the United Nations.

## Background

Tuberculosis (TB) remains a serious public health issue, especially in the low- and middle-income countries. Globally, there were an estimated 9.6 million new TB cases and 1.5 million TB deaths in 2014 [1]. According to the Global Tuberculosis Report 2015, effective diagnosis and treatment have saved 43 million lives between 2000 and 2014 [1]. However, the spread of multiple drug resistant tuberculosis (MDR-TB) is undermining global TB control. It was estimated that 3.3 % new cases and 20 % previously treated cases had MDR-TB; and in 2014, nearly 190 000 cases died from MDR-TB [1]. Resistant strains emerged mainly from the inadequate use of TB drugs, use of low-quality drugs, poor TB program performance or lack of regulation [2]. The currently recommended treatment for new cases of drug-susceptible TB is a six-month regimen of four first-line drugs: isoniazid, rifampicin, ethambutol, and pyrazinamide. While MDR-TB patients are eligible for second-line treatment. To reduce this burden, treatment coverage gaps must be addressed with adequate and quality supplies for both first-line and second-line drugs.

As one of the 22 high TB burden countries, China has made great progress by reducing 51 % of TB prevalence and 79.5 % of mortality rate between 1990 and 2010, achieving the TB control targets of the United Nation’s Millennium Development Goals ahead of time [3]. This could be attributed to the nationwide implementation of the World Health Organization (WHO)-recommended DOTS (directly observed treatment, short-course) strategy since the 1990s. However, China still has the world second highest burden of both TB and MDR-TB in 2014. It was estimated that the prevalence and incidence of TB in China were 668 and 390 per 100 000 population, respectively. Meanwhile, 5.7 % new TB cases and 26 % retreated cases were MDR-TB in China [1]. This was mostly because of the inappropriate TB treatments and treatment interruption, especially among patients treated within the hospital system. TB treatment in China is provided by CDC and hospitals. Hospitalization for 100 % of MDR-TB patients in 2014 was reported in China [4]. However, hospitals provide limited outpatient follow-up of TB patients. A Chinese survey showed that over 40 % of the MDR-TB patients did not complete their last treatment course, among whom most were treated within the hospitals [5]. The Chinese government has taken many actions along with DOTS, such as the free treatment policy and the use of anti-TB fixed-dose combination (FDCs). Studies are needed to illustrate the effect of policies and regulations for TB prevention in China, and to provide new ideas for TB control. Until recently, no study was seen to use the production and sales data to evaluate this issue.

Understanding the production and sales status of anti-TB drugs in China could also provide the world a glance of how China might contribute to global TB control by drug supply. The total market size of anti-TB drugs in low-and middle-income countries is around $730 million. Experts expected that the market for first-line drugs would remain stable in the future while second-line drug market would continue to expand with the improvement of detection technology for drug-resistant TB. Research on the anti-TB drug market is needed, however, only few studies were seen, especially in the perspective of the influence of policies. Limited literatures on developing countries such as Indian, Brazil, Philippines have revealed that different countries face different patterns of the anti-TB drug market [6–8]. Since many countries are still lack of TB treatments, China is expected to contribute to global TB control through drug supplies. However, no paper has discussed this possibility through quantitative production and sales data. Only few market analyses were done many years ago in China, focusing on limited drug types [9–11].

Thus, this study analyzed the recent production and sales situation of anti-TB drugs to reveal the current market of anti-TB drugs in China, and to discuss the possible influence of policy and regulation. This research could not only help value China’s production capacity and medication situation towards TB treatment, but also reveal the possibility for China to better participate in global TB control.

## Methods

### Data sources

The production data of anti-TB drugs in China from 2011 to 2013 were from the Chinese government statistics reported via the Ministry of Industry and Information Technology (MIIT). MIIT requires certain manufacturing enterprises to self-report their production data each year. This data was the official national database of domestic production in China. However, due to the limitation of self-report, data on pyrazinamide active pharmaceutical ingredients (APIs) production was missing and not included in this analysis.

The original sales data of China’s anti-TB drugs during 2010 and 2014 were provided by IMS China from IMS China Hospital Pharmaceutical Audit (IMS CHPA) data source. IMS CHPA is the sampling statistics from over 9 000 hospitals in China and then zoom to the national level reflecting the drug purchasing situation in hospitals with 100 or more beds. IMS is a data source that includes sales data from various countries using the standard data collecting protocol. It has been used for country comparisons in research articles [12].

### Data analysis

Anti-TB drugs were classified into APIs and finished drugs in this research. APIs were classified statistically in the molecular level while finished drugs were not only classified in the molecular level but also according to the components of the specifications of preparation. The classification of first-line and second-line anti-TB drugs was also used during analysis. The main first-line drugs studied in this research were rifampicin, isoniazid, pyrazinamide, ethambutol, and streptomycin.

SAS package was used to group the original data, and to extract and unify the molecular level of the key components. The production and sales weight of the drugs were then calculated by using the specifications of preparation and packing. All the key components were summed into different types of sublists. Microsoft Excel was used for analysis and description.

## Results

### The production status of anti-TB drugs in China

**Fig. 1:**
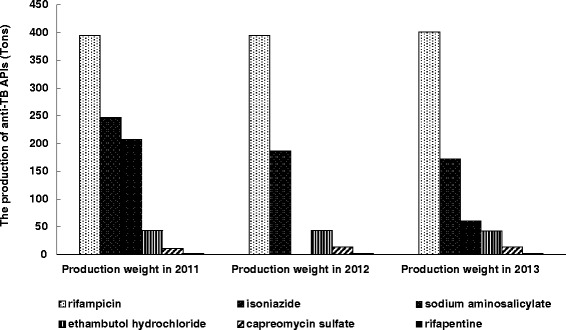
The change of production weight of the main anti-TB APIs in China between 2011 and 2013. Due to the limitation of data collection, data on pyrazinamide APIs production was missing and not included in this analysis. No data was found on sodium aminosalicylate in 2012

After adding the available production weight of anti-TB APIs during 2011 and 2013, we found that basic anti-TB APIs – rifampicin (1 191 tons) and isoniazid (606 tons) had the highest production, followed by sodium aminosalicylate, ethambutol hydrochloride, capreomycin sulfate and rifapentine (Fig. 1). Anti-TB APIs in China were mainly first-line drugs, while the second-line drug sodium aminosalicylate also showed a high production of 269 tons between 2011 and 2013.

**Fig. 2:**
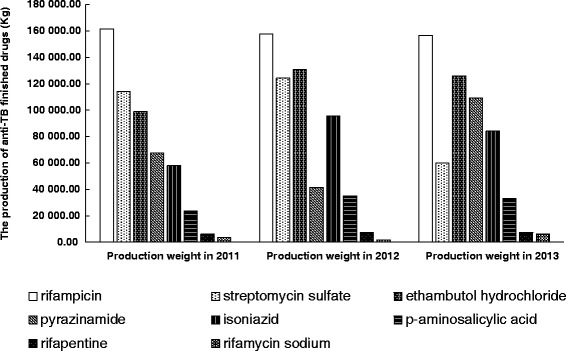
The change of production weight of the main anti-TB finished drugs (molecular level) in China between 2011 and 2013

Finished drugs were classified into the molecular level to analyze drug production status. The top five production weight of anti-TB finished drugs in 2013 was rifampicin, ethambutol hydrochloride, pyrazinamide, isoniazid, and streptomycin sulfate, which were all first-line drugs. The production weight of first-line finished drugs takes up to 93, 92 and 92 % of the total finished drugs in 2011, 2012 and 2013, respectively (Fig. 2). The total trend of anti-TB finished drugs in China was rather stable between 2011 and 2013. However, the production of streptomycin sulfate in 2013 was less than 50 % of that in 2012, which decreased from 124 360 kg to 59 911 kg.

Though the production of anti-TB FDCs in China was still quite small, an increasing trend was observed between 2011 and 2013. The total production weight of FDCs rose rapidly from 10 tons in the year of 2011 to 60 tons in 2012 and reached 99 tons by 2013. The number of total production pieces also tremendously increased from 80 million in 2011 to 350 million in 2013.

### The sales status of anti-TB drugs in China

**Fig. 3:**
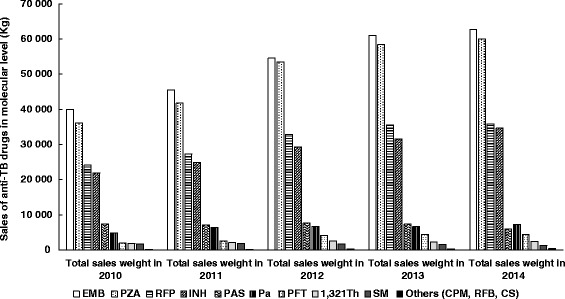
The change of sales weight of the main anti-TB drugs (molecular level) in China between 2010 and 2014. CPM, capreomycin; CS, cycloserine; EMB, ethambutol; INH, isoniazid; Pa, pasiniazid; PAS, p-aminosalicylic acid; PZA, pyrazinamide; RFB, rifabutin; RFP, rifampicin; RFT, rifapentine; SM, streptomycin; 1 321Th, protionamide

The total top four sales of anti-TB drugs in China during 2010 and 2014 were ethambutol (263.75 tons), pyrazinamide (249.85 tons), rifampicin (155.59 tons), and isoniazid (142.42 tons), which were far more than the rest of anti-TB drugs. Except for streptomycin, the sales weight of first-line drugs in the molecular level all showed a tendency of increase with around 10 % growth rate annually from 2010 to 2014 (Fig. 3). The sales weight of streptomycin continuously declined since 2012, from 1695 kg in 2012 to 1216 kg in 2014. While for the sales weight of the second-line drugs, they all rose annually expect for p-aminosalicylic acid. In total, first-line anti-TB drugs accounted for nearly 90 % of the anti- TB finished drug market each year (Table 1).

**Table 1:** Total sales weight of first-line and second-line anti-TB drugs in China from 2010 to 2014 (Kg)

The total sales weight	2010	2011	2012	2013	2014
First-line drugs	123 931	141 158	172 034	188 051	194 446
Second-line drugs	16 103	18 163	21 065	20 831	20 415
The percentage of first-line drugs in anti-TB drugs	89 %	89 %	89 %	90 %	90 %

The sales of the drugs for initial pulmonary TB treatment took up to 53, 56, 63, 67 and 71 % of all anti-TB drugs, while the volume for retreated pulmonary tuberculosis accounted for 9, 9, 7, 6 and 4 % in the year of 2010, 2011, 2012, 2013 and 2014, respectively. The rest proportion was for treating MDR-TB and those not mentioned in China’s *Standard for Diagnosis and Treatment of Pulmonary Tuberculosis (2012 Edition).*

In the past 5 years, sales of anti-TB drugs rose annually, especially for the single ingredient medicines. The total sales volume of single ingredient medicines increased 35.2 % between 2011 and 2014, and accounted for 95.7 % of all anti-TB drugs in 2014. While the percentage of sales volume for FDCs, though slightly increased, only account for less than 2 % of the total sales volume each year.

## Discussion

This study showed that the production and sales of the first-line anti-TB drugs have been increasing despite the falling TB prevalence in China; while for those that were removed from the first-line drug list or not listed as first-line drugs, the number has been fallen in both production and sales. The low production and sales of anti-TB FDCs and the second-line TB drugs rose concerns for the proper and adequate treatment of MDR-TB. The redundant production amount suggested a possibility for China to contribute to global TB control through drug supplies.

The result showed that the production of anti-TB FDCs in China were increasing. The use of FDCs can help to reduce incorrect medication, avoid improper single-drug regimen and secondary drug resistance [13]. Not much attention was paid in this area before resulting in the rapid increase of MDR-TB cases. From 2008, guidelines and pilot programs have been conducted by the Chinese government responding to WHO’s call to promote the use of FDCs in the TB treatment. As mentioned in the Supply and Management of Anti-TB Drugs of *Guidelines for Implementing the National Tuberculosis Control Program in China (2008)*, anti-TB FDCs deserve popularization in areas with the necessary conditions [14]. Since 2008, pilots on the use of anti-TB FDCs have been launched in 11 provinces (autonomous regions and municipalities). As the result of the impletion of policies and guidelines, by the end of 2010, 10 % of patients with pulmonary tuberculosis have used anti-TB FDCs and 17 drug companies have been qualified to manufacture anti-TB FDCs [15]. In accordance with these changes, this study also illustrated the increase in production of anti-TB FDCs in China after 2011. However, China is still in the early stage of using anti-TB FDCs, with the small number of varieties and the low proportion of sales.

This study showed that the production and sales of first-line anti-TB drugs have been increasing despite the falling prevalence. This might be related to the free anti-TB drug treatments for new active pulmonary tuberculosis cases and retreated smear positive pulmonary tuberculosis patients offered by the Chinese government [11]. The results also revealed that first-line anti-TB drugs held absolute superiority, taking up around 90 % of the production and sales of the domestic anti-TB drug market. The recommended medicines in *Standard for Diagnosis and Treatment of Pulmonary Tuberculosis (2012)* for initial and retreated pulmonary tuberculosis both include the combination of isoniazid, rifampicin, pyrazinamide and ethambutol, which were all on the top of our sales list. It can be inferred that the sales market of anti-TB drugs in China is matched with the medication regimens recommended by guidebooks. With the standardization of treatment and the free treatment policy, there should be no surprise that the production and sales of first-line anti-TB drugs have been increasing despite the falling prevalence.

Though adequate first-line drugs can be provided through the system, improper use of these drugs has increased the prevalence of MDR-TB in China. According to China’s National Survey of Drug-Resistant Tuberculosis in 2007, one in four TB patients treated in the public health system were receiving first-line tuberculosis drugs while they have the disease that is resistant to first-line isoniazid or rifampin (or both) [5]. Addressing MDR-TB in China requires more emphasis on second-line anti-TB treatment. However, the low production and sales of the second-line drugs rose concerns for the treatment of MDR-TB. Second-line anti-TB drugs are included in the WHO-recommended regimen for MDR-TB treatment. Globally, around 90 % of detected MDR-TB patients in 2014 had started second-line treatment. Though China has reported 100 % hospitalization of MDR-TB patients in 2014, the limited outpatient follow-up of TB patients causes the incompletion of MDR-TB treatment and the improper use of first-line drugs [5]. Relevant policies are needed in China to focus more on this issue.

Our results indicated that national policies might have the ability to provide prompt influence on anti-TB drugs. Drugs that were removed from or not listed in the first–line drug list have shown a fallen trend in both production and sales. For example, due to the severe side-effects and the high rates resistance in drug-resistant TB, streptomycin was removed from the list of first-line drugs and is no longer in the major treatment plans for retreated patients in the treatment guidelines of WHO and China [15–17]. As a result, a rapid decrease in the production and sales of streptomycin has been noticed since 2012. Another example is the relatively small sales volume of rifapentine and rifabutin in China. Rifapentine and rifabutin are both the first-line drugs recommended by WHO, however, they were both not listed as main treatment drugs in China’s official guidelines. This again suggested the possible influence of national guidelines on the production and sales of anti-TB drugs in China.

Although this study used currently the most accurate and official data, the source of the data still face some limitations. First, since the production data were self-reported to the Chinese government by certain enterprises, the data might be misstated, omitted or merged. Also, the production weight of finished products or FDCs might include the weight of other partner drugs or packaging. However, due to the stable standard requirements, the results should illustrate the actual tendency. Since this data was the only governmental official data and used for policymaking, the results should provide enough information for further discussion. Besides, the sales data from IMS CHPA only reflects the drugs sold in hospitals with 100 or more beds. Though most patients receive treatment from these hospitals, the data might be smaller than the actual number, since medicines sold from other places were not included.

## Conclusion

This study showed the macro picture of the production and sales situation of anti-TB drugs in China. Despite the falling prevalence in China, with the standardization of treatment and the free treatment policy, the production and sales of first-line anti-TB drugs have been increasing. The limited amount of second-line anti-TB drugs and FDCs rose concerns for the adequate treatment of MDR-TB, and should be noticed for future policymaking. Policies, guidelines, plans and standards from the national government and WHO might place important influences on the production and sales of anti-TB drugs, which in turn contribute to TB control and treatment. Moreover, the redundant production amount also illustrated the possibility for China to contribute to global TB control through drug supplies.

## Additional file

Additional file 1: Multilingual abstracts in the six official working languages of the United Nations. (PDF 725 kb)

## Abbreviations

1,321Th: Protionamide
APIs: Active pharmaceutical ingredients
CPM: Capreomycin
CS: Cycloserine
DOTS: Directly observed treatment, short-course
EMB: Ethambutol
FDCs: Fixed-dose combination
INH: Isoniazid
MDR-TB: Multiple drug resistant tuberculosis
MIIT: Ministry of industry and information technology
Pa: Pasiniazid
PAS: p-aminosalicylic acid
PZA: Pyrazinamide
RFB: Rifabutin
RFP: Rifampicin
RFT: Rifapentine
SM: Streptomycin
TB: Tuberculosis
WHO: World Health Organization
