# Supplementary material for: The production and sales of anti-tuberculosis drugs in China
Source: Infect Dis Poverty. 2016 Oct 4;5:88. doi: 10.1186/s40249-016-0184-z (PMC5048613; doi:10.1186/s40249-016-0184-z)
Supplement: Additional file 1: — Multilingual abstracts in the six official working languages of the United Nations. (PDF 725 kb) [file 40249_2016_184_MOESM1_ESM.pdf]

Translation of the abstract into the six official working languages of the United Nations

إنتاج أدوية العلاج من السل ومبيعاتها في الصين

يانغ مو هوانغ، كي بينغ زاو، كياو مينغ رين، دان لو بينغ، يان غاو

الملخص
مقدمة: يعتبر السل أحد الأمراض المعدية الكبرى عالمياً. ويشكل الاستخدام المناسب والملائم لأدوية العلاج من السل سبباً رئيسياً في التحكم في هذا المرض. تهدف هذا الدراسة إلى دراسة السعة الإنتاجية لأدوية العلاج من السل وحالة مبيعاتها في الصين، وإلى تناول إمكانية مساهمة الصين في السيطرة العالمية على مرض السل.
منهج البحث: تم استخلاص البيانات المتعلقة بإنتاج أدوية العلاج من السل في الصين بين عامي 2011 و2013 وبيانات مبيعاتها بين عامي 2010 و2014 من قاعدة بيانات وزارة الصناعة وتكنولوجيا المعلومات وقاعدة بيانات آي إم إس للرعاية الصحية، على التوالي. تم ضبط عدد الأدوية بالمستوى الجزيئي لعناصرها الأساسية قبل البدء في العملية الحسابية، كما تم وصف البيانات وتحليلها بواسطة برنامج مايكروسوفت إكسل.
النتائج: أظهرت النتائج أن أدوية الخط الأول تملك الحظ الأكبر من المبيعات (89.5%) والإنتاج (92.3%) لأدوية العلاج من السل في الصين. ووجد أن دواء ريفامبيسين يحتوى على أكبر كمية من المكونات الصيدلانية النشطة والمنتجات تامة الصنع، بينما وجد أن عقاري إيثامبيوتول وبيرازيناميد قد حققا الدرجة الأولى والثانية في مبيعات المنتجات تامة الصنع. وتشكل المركبات ذات الجرعة الثابتة بحسب النتائج نسبة ضئيلة من الحجم الإجمالي للإنتاج والمبيعات، مع زيادة بسيطة فقط. وأظهرت نتائج الإنتاج والمبيعات لدواء ستريبتوميسين انخفاضاً ثابتاً بعد عام 2012. وبشكل عام، فقد ظهر أن اتجاهات وحصص الأنواع المختلفة لعلاج السل تتشابه في الإنتاج والمبيعات، إلا أن حجم الإنتاج كان أكبر بشكل ملحوظ من حجم المبيعات، وبشكل خاص لعقاري ريفامبيسين وآيزونيازيد.
الخلاصة: خلصت هذه الدراسة إلى أن أدوية الخط الأول تتبوأ الرتبة الأولى بين الأدوية التي يتم إنتاجها واستعمالها في الصين. ومن الناحية الأخرى، فإن الحجم المنخفض في إنتاج ومبيعات أدوية العلاج من السل في الخط الثاني والمركبات ذات الجرعة الثابتة يشكل قلقاً من ناحية علاج السل المقاوم للأدوية المتعددة. وتدل الكميات الإنتاجية المتكررة إلى جانب التأثير المباشر للسياسة الوطنية على إنتاج الأدوية ومبيعاتها إمكانية للصين في المساهمة في السيطرة العالمية على مرض السل بشكل أفضل.

Translated from English version into Arabic by Maysa Orabi, through

TRANSLATORS
WITHOUT BORDERS

中国抗结核药物的生产和销售现状分析

黄旸木, 赵启鹏, 任巧萌, 彭丹璐, 郭岩

摘要
引言：结核病是全球主要传染病之一。足量和恰当地使用抗结核药物是结核病防控的关键。本研究旨在分析中国抗结核药物的生产能力和销售现状，并进一步探讨中国参与全球结核病防控的可能性。
方法：中国抗结核药物 2011 年至 2013 年的生产数据及 2010 年至 2014 年的销售数据分别从中国工信部数据库和艾美仕数据库获得。销售和生产数据根据药物分子名级别的核心成分标化计算。所有数据采用 Excel 软件进行描述和分析。
结果：中国抗结核药品的销售（89.5%）和生产（92.3%）均以一线药为主。原料药和成品药生产以利福平为主，成品药销售以乙胺丁醇和吡嗪酰胺为主。固定剂量复合制剂占抗结核药物总生产和销售重量的比例小，但呈上升趋势。链霉素的生产和销售自 2012 年以来呈下降趋势。中国抗结核药物的生产和销售趋势相似，但生产重量远大于销售量，以利福平和异烟肼最为突出。
结论：中国生产和使用的主要抗结核药物为一线药。二线抗结核药物和固定剂量复合制剂的低生产量和销售量，可能影响对多耐药结核病的治疗。中国充足的抗结核药生产量和国家政策对该类药物生产和销售的迅速影响提示，中国有潜力为全球结核病防控做出更多贡献。

Translated from English version into Chinese by Yang-Mu Huang

La production et la vente de médicaments anti-tuberculeux en Chine

Yang-Mu Huang, Qi-Peng Zhao, Qiao-Meng Ren, Dan-Lu Peng, Yan Guo

Résumé

Contexte: la tuberculose (TB) constitue une importante maladie infectieuse dans le monde entier. L'administration adéquate et appropriée de médicaments anti-tuberculeux est essentielle à la lutte contre la tuberculose. La présente étude consiste à évaluer les capacités de production et les ventes de médicaments anti-tuberculeux en Chine et d'aborder le potentiel de la Chine à contribuer à la lutte mondiale contre la tuberculose.
Méthodes: les données de production des médicaments anti-tuberculeux en Chine de 2011 à 2013 et les données de vente de 2010 à 2014 ont été respectivement extraites de la base de données du Ministère chinois de l'Industrie et des Technologies de l'information et de la base de données de l'entreprise IMS Health. Le nombre de médicaments a été standardisé à l'échelle moléculaire des principaux composants avant le calcul. Toutes les données ont été décrites et analysées à l'aide du programme Microsoft Excel.
Résultats: les médicaments de première ligne représentaient la majorité des médicaments anti-tuberculeux vendus (89,5 %) et produits (92,3 %) en Chine. La production de la rifampicine constituait la majorité des principes pharmaceutiques actifs (PPA) et des produits finis, tandis que l'éthambutol et la pyrazinamide constituaient les deux principaux produits finis vendus. Les combinaisons à dose fixe ne représentaient que de petits pourcentages en termes de production totale et de pondération des ventes, bien que l'on ait constaté une légère hausse. La production et la vente de la streptomycine ont présenté une tendance à la baisse après 2012. Les tendances et la proportion de différents médicaments anti-tuberculeux étaient similaires en termes de production et de vente, bien que la pondération en fonction de la production était plus nette que celle des ventes, notamment pour la rifampicine et l'isoniazide.
Conclusions: les médicaments de première ligne constituaient les principaux médicaments produits et utilisés en Chine. Le traitement de la tuberculose multirésistante aux médicaments est préoccupante face à la faiblesse de la production et des ventes de médicaments anti-tuberculeux de deuxième ligne et de combinaisons à dose fixe. La quantité de production superflue, ainsi que l'influence rapide de la politique nationale sur la production et les ventes des médicaments ont démontré le potentiel d'augmentation de la contribution de la Chine à la lutte mondiale contre la tuberculose.

Translated from English version into French by eric ragu, through

TRANSLATORS
WITHOUT BORDERS

Производство и продажа противотуберкулезных препаратов в Китае

Янг-Му Хуанг, Куи-Пенг Жао, Квьяо-Менг Рен, Дан-Лу Пенг, Йан Гуо

Резюме
Краткая информация: Туберкулез (ТВ) является серьезным инфекционным заболеванием, распространенным во всем мире. Обоснованный прием противотуберкулезных препаратов необходим для контроля за ТВ. Данное исследование посвящено изучению потенциала Китая в области производства и продаж противотуберкулезных препаратов, а также обсуждению возможностей Китая по вкладу в достижение глобального контроля за распространением ТВ.
Методы: Данные о производстве противотуберкулезных препаратов в Китае за 2011-2013 годы и о продажах за 2010-2014 получены от Министерства промышленности и информационных технологий и службы по здравоохранению ММС, соответственно. Перед анализом препараты были сгруппированы по ключевым компонентам. Вся информация была описана и проанализирована с помощью Microsoft Excel.
Результаты: Препараты первой линии составили большинство и в объеме продажах(89,5 %) и в производстве (92,3 %) противотуберкулезных средств в Кита. Наибольшую долю среди активных фармацевтических субстанций (АФС) и готовых препаратов занимает производство рифампицина, в то время как этамбутол и пиразинамид возглавили список продаж готовых препаратов. В общей массе произведенных и проданных препаратов комбинированные препараты с фиксированной дозировкой занимают малую долю, хотя и с небольшой тенденцией к увеличению. Производство и продажа стрептомицина с 2012 года снизились. Динамика и количество производства и продаж различных противотуберкулезных препаратов были схожи, в то время как объем производства превысил объем продаж, в особенности это относится к рифампицину и сониазиду.
Выводы: Препараты пбервой линии занимают доминирующее место среди произведенных и потребляемых препаратов в Китае. В то же время, низкий уровень производства и продаж препаратов второй линии и комбинированных препаратов с фиксированной дозировкой вызывает обеспокоенность в плане лечения лекарственно-резистентного туберкулеза различных форм. Избыточное производство, а

также грамотная национальная политика в области производства и продаж обеспечивают потенциал Китая по установлению глобального контроля над распространением туберкулеза.

Translated from English version into Russian by Ms Zhdanova, through

TRANSLATORS
WITHOUT BORDERS

Producción y venta de medicamentos antituberculosis en China

Yang-Mu Huang, Qi-Peng Zhao, Qiao-Meng Ren, Dan-Lu Peng, Yan Guo

Resumen

Antecedentes: La tuberculosis (TB) es una seria enfermedad infecciosa a nivel mundial. Para su control, es fundamental el uso adecuado y correcto de los medicamentos antituberculosis. El objetivo del presente estudio es estudiar la capacidad de producción y la situación de las ventas de medicamentos antituberculosis en China, y analizar más a fondola capacidadde China de contribuir al control mundial de la TB.

Métodos: Los datos sobre la producción de medicamentos antituberculosis en China entre 2011 y 2013 y las ventas entre 2010 y 2014 se extrajeron de la base de datos del Ministerio de Industria y Tecnología de la Información de China y de la base de datos del instituto IMS Health, respectivamente. Antes de realizar los cálculos se estandarizó la cantidad de medicamentos al nivel molecular de los componentes clave. Toda la información se describió y analizó con Microsoft Excel.

Resultados: Los medicamentos de primera línea constituyeron la mayoría de los medicamentos antituberculosis en China tanto en ventas (89,5%) como en producción (92,3%). La producción de rifampicina tuvo el mayor porcentaje de ingredientes farmacéuticos activos y productos terminados, mientras que el etambutol y la pirazinamida fueron los dos mejores en ventas en productos terminados. Las combinaciones en dosis fija solo representaron un pequeño porcentaje en el peso total de producción y ventas, a pesar de que se observó un pequeño incremento. La producción y venta de estreptomicina mostró una tendencia descendente después del año 2012. Las tendencias y la proporción de los distintos medicamentos antituberculosis fueron similares en producción y en ventas, pero el peso de la producción fue mucho mayor que el de las ventas, en particular para la rifampicina y la isoniacida.

Conclusiones: Los medicamentos de primera lína fueron los principales medicamentos producidos y utilizados en China. Mientras que la menor producción y venta de los medicamentos antituberculosis de segunda línea y los medicamentos en dosis fija fueron motivo de preocupación para el tratamiento de la TB resistente a múltiples medicamentos. La cantidad redundante de producción, al igual que la influencia pronta de las políticas nacionales sobre la producción y venta de medicamentos, indicaron que China tiene potencial para ser un mejor contribuyente en el control de la TB a nivel mundial.

Translated from English version into Spanish by Maria Alejandra Aguada, through

TRANSLATORS
WITHOUT BORDERS
